# A Rare Cause of Dyspnea: Sudden Rupture of Aortic Valsalva Sinus Aneurysm

**DOI:** 10.1155/2013/909302

**Published:** 2013-02-27

**Authors:** Erdinç Arıkan, Arif Karagöz, Serdar Bayata, Levent Yilik, Erden Erol Ünlüer

**Affiliations:** ^1^Department of Cardiology, İzmir Katip Celebi University Atatürk Research and Training Hospital, 35360 Karabağlar, İzmir, Turkey; ^2^Department of Emergency Medicine, İzmir Katip Celebi University Atatürk Research and Training Hospital, 35360 Karabağlar, İzmir, Turkey; ^3^Department of Cardiovascular Surgery, İzmir Katip Celebi University Atatürk Research and Training Hospital, 35360 Karabağlar, İzmir, Turkey

## Abstract

Aneurysm of the sinus of Valsalva is an uncommon cardiac abnormality; however, the most common complication is rupture into the right heart chambers or rarely towards the left chambers. A ruptured aneurysm typically leads to an aortocardiac shunt and progressively worsening heart failure. We report a case of a 21-year-old male who suffered an aneurysm of the sinus of Valsalva rupture into the right atrium who underwent successful surgical repair.

## 1. Introduction

Aneurysm of the aortic sinus, also known as the sinus of Valsalva (SV), is rare. When present, it is usually either in the right or in the noncoronary sinus, rarely in the left sinus [1, 2]. An aortic sinus is one of the anatomic portions of the ascending aorta, which lies just above the aortic valve. There are three aortic sinuses: the left anterior, the right anterior, and the posterior. This type of aneurysm is typically congenital and may be associated with heart defects. It is sometimes associated with Marfan syndrome or Loeys-Dietz syndrome but may also result from Ehlers-Danlos syndrome, atherosclerosis, syphilis, cystic medial necrosis, chest injury, or infective endocarditis.

If unruptured, it may be asymptomatic and therefore go undetected until symptoms appear or radiologic imaging is performed for other reasons [3]. We present a case of ruptured congenital aortic sinus aneurysm that was diagnosed using two-dimensional echocardiography and aortography. 

## 2. Case Story

**Figure 1:**
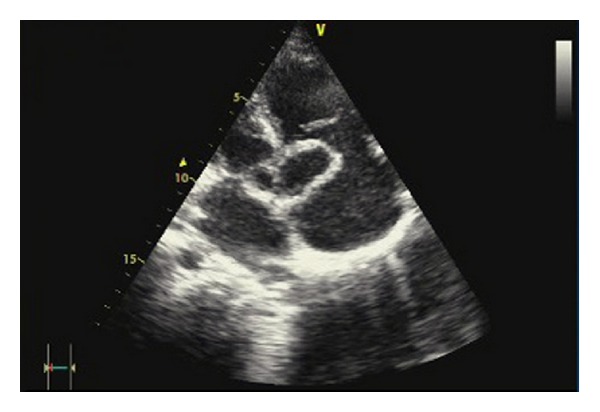
Sinus Valsalva aneurysm in apical five-chamber view.

**Figure 2:**
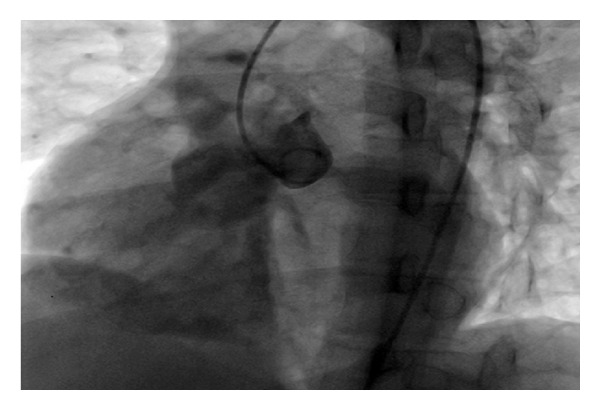
Opacification of right ventricle during angiography of aortic root.

A 21-year-old male was brought to the Emergency Department (ED) with acute onset dyspnea and hypotension. He was performing military service as a private and his friends reported nothing remarkable about his health until he suddenly collapsed that evening. His first physical exam in the ED revealed poor general condition with unpalpable pulses, profuse diaphoresis, and lethargy. Heart rate was 120 bpm, and blood pressure was 70/30 mmHg. Jugular venous distention was present. He had normal lung sounds, and a systolodiastolic murmur was audible in the mesocardiac region. ECG showed sinus tachycardia with normal axis. Arterial blood gas analysis revealed oxygen desaturation with respiratory alkalosis. He was initially evaluated for acute pulmonary embolism, which was excluded with a pulmonary CT angiogram. The on-call cardiology consultant performed an emergency echocardiogram (ECHO). The ECHO revealed a ring-like image in the right atrium (RA) in the apical four-chamber view. In modified views, this ring-like image was found to be a protrusion of the SV to the RA at the level of the tricuspid valve (Figure 1). In color Doppler, there was a high velocity jet directly to the atrial side of the tricuspid through a 2 mm orifice. The patient was immediately transferred to the catheterization laboratory for confirmation of pathology and additional abnormalities. The aortogram just above the aortic valves was the only image that was taken that was sufficient to demonstrate the gross regurgitation of the contrast from the sinus of the Valsalva aneurism (SVA) to the right atrium and the right ventricle (Figure 2). There was no valvular aortic regurgitation to the left ventricle. The aortic diameter was measured as 41 mm at the level of the SV and 31 mm at the tubular portion. The patient underwent emergency surgery for the repair of the aortic root. After midline sternotomy, on inspection the right cardiac chambers were extensively dilated and edematous. Under cardiopulmonary bypass (CPB), a 2 × 1 cm tear was discovered right above the commissure of the right and non-coronary cusps, interconnecting the aorta to the right atrium. A right atriotomy was made, and the defect that opened slightly superior to the tricuspid valve was primarily repaired with 4/0 prolene. A patch plasty was performed for the defect on the aortic side using a Dacron graft and 4/0 prolene. After entrainment of the right heart, CPB was successfully terminated with full inotropic support (cross clamp time: 98 min; CPB time: 192 min). As the heart was extremely edematous and swollen, the sternum was left open, and skin and subcutaneous tissues were sutured closed. The sternum was closed uneventfully after 24 hours. The patient was discharged on postoperative day seven. On the outpatient follow-up, he was in good health at three months. 

## 3. Discussion

Sinus of Valsalva aneurysm is uncommon and is encountered in 0.14–0.96% of open heart surgery cases. The incidence of SVA is reported to range between 0.1 and 3.5% of all congenital heart defects [2]. An autopsy series of 8138 individuals suggests a prevalence of 0.09% in the general population [3]. SVA has a marked male preponderance (4 : 1), and the incidence is higher in Asian populations [4]. Ruptured aneurysms are most common in men and in the third or fourth decade of life [5]. In 1919, Abbott clearly established a congenital etiology for SVA [6]. Acquired SVA occurs less frequently and is caused by conditions affecting the aortic wall, such as infection (syphilis, bacterial or fungal endocarditis, and tuberculosis), degenerative disease (atherosclerosis, connective tissue disorders, and cystic medial necrosis), or thoracic trauma [7, 8]. Unruptured SVA is usually asymptomatic. Currently, with the noninvasive imaging modalities such as echocardiography and MRI, they are more frequently diagnosed. Exertional dyspnea, palpitations, and angina-like chest pain were reported in patients with unruptured SVA [9]. SVA is usually diagnosed in the setting of a rupture. Most ruptures develop between 20 and 40 years of age. Electrocardiograms usually show voltage criteria for left ventricular hypertrophy and ST-T wave abnormalities. The consequences of rupture depend on the size and rapidity of the process leading to rupture. In one-third of patients, left-to-right shunting immediately following the rupture of an aneurysm into the right side of the heart produces acute dyspnea and chest pain. However, half of patients note gradually worsening dyspnea, fatigue, chest pain, and peripheral edema over several months or even years following rupture, and the remainder of patients are still asymptomatic at the time of diagnosis [10]. Our patient presented with dyspnea and eventually hemodynamic collapse. Ruptured aneurysm to the right atrium was confirmed by echocardiography and aortography. Clinical diagnosis of a ruptured SVA may be difficult. Sinus of Valsalva aneurysms may cause continuous murmurs when they rupture into the right heart. In almost all cases, rupture occurs from the right or noncoronary SV into the right heart chambers. The murmur is heard most loudly at the lower sternal edge or xiphoid. Diastolic accentuation of this murmur is an important sign of differentiated ruptured SVA from patent ductus arteriosus. Low diastolic blood pressure is another clinical finding. Clinical diagnosis of ruptured SVA can easily be confirmed by echocardiography. Two-dimensional echocardiography with color flow provides an accurate noninvasive means of demonstrating the aneurysm itself and the left to right shunting following rupture. Asymmetric dilatation of the aortic root by SVA can easily be visualized. Doppler echocardiography in the setting of ruptured SVA to the right heart chambers demonstrates fluttering of the tricuspid valve leaflets, a color jet from the aortic root into the right atrium or ventricle, and diastolic opening of the pulmonary valve. Surgical correction is indicated on an emergency basis after confirmation by echocardiography and cardiac catheterization, if needed. If treatment is given as early as possible, the development of congestive heart failure, infective endocarditis, and mortality will be reduced. The rupture of SVA is a condition that can be confused with pulmonary embolism and can cause catastrophic consequences with unnecessary thrombolytic administration. Aortic dissection and myocardial infarction should also be considered in differential diagnosis of ruptured SVA.
